# Rethinking planetary protection: an island biogeographical analysis

**DOI:** 10.1098/rsif.2025.0079

**Published:** 2025-06-25

**Authors:** Daniel J. Brener, Charles S. Cockell

**Affiliations:** ^1^ Higgs Centre for Theoretical Physics, University of Edinburgh, Edinburgh, UK; ^2^ UK Centre for Astrobiology, School of Physics and Astronomy, University of Edinburgh, Edinburgh, UK

**Keywords:** planetary protection, biogeography, interplanetary, life, population dynamics

## Abstract

We reconsider the problem of planetary protection using, by the analogy of planets as islands, the theory of island biogeography. We show that although the notion of equilibrium populations that emerge from the effects of immigration and extinction generally breaks down when applied to interplanetary scales, the mean-time to extinction resulting from the combined effects of growth and death rates can be quantified. We reconsider the probabilistic model of planetary protection, discuss how mean-time to extinction can instead be used to assess contamination risk, and we propose a research direction for planetary protection based on these ideas. We discuss more broadly the applicability of island biogeography to considering biotic transfer at interplanetary scales.

## 1. Introduction

Can life be successfully transferred between planets? This question has been explored in both natural contexts (panspermia) [1] and human-aided transfer, whether deliberate [2] or unintentional. The potential for unintentional transfer has led to discussions on planetary protection [3,4], focusing on the risk of contaminating other planetary environments (forward contamination) and the potential harm to Earth’s biosphere from extraterrestrial life (back contamination).

Numerous attempts have quantified the factors involved in interplanetary life transfer. In planetary protection, the favoured approach is probabilistic, assigning probabilities to organisms’ survival and growth in various transfer phases, such as surviving the interplanetary journey and successful growth in the destination environment. This approach can place quantitative numerical limits on microbial loads, for example, on specific missions [5]. For instance, the Committee on Space Research (COSPAR) policy on planetary protection, followed by NASA [6], directs that the probability that a planetary body will be contaminated during any one exploratory mission to Mars shall be no more than $\text{1}\times {\text{10}}^{-3}$ [7]. In the case of the Europa Clipper mission, it was estimated that the probability of contaminating the moon was $\text{2.25}\times {\text{10}}^{-5}$ [5].

The most common method for calculating these probabilities is the Coleman–Sagan equation, which gives the probability of contamination, $P_{c}$:


(1.1) $$P_{c}=n_{0}RP_{s}P_{I}P_{R}P_{g},$$

where $P_{s}$, $P_{I}$, $P_{R}$ and $P_{g}$ are the probabilities that the microorganisms reach the planetary surface, the spacecraft impacts the planet, the microorganisms are released to the environment post-landing and for growth, respectively, and $n_{0}$ and R are the number of microorganisms initially on the spacecraft and their rate of reduction due to conditions on the spacecraft before and after launch, respectively [8]. The terms within this approximation capture the same phases of transfer that can be estimated for the natural transfer of life between planets [1].

However, critiques of the probabilistic method have emerged, notably in the National Academies of Sciences (NAS) report on planetary protection for icy solar system bodies [9]. The NAS report highlighted substantial limitations of probabilistic models, such as uncertainties in the magnitude of bioload reduction factors and the problematic assumption that individual contamination factors are independent and can be estimated with sufficient statistical robustness to be useful in prediction.

Probabilistic parameters provide quantitative bounds on the chance of an organism reaching a planet. However, once the organism is there, the chance of colonization is not probabilistic, but rather determined by whether the environment is suitable for replication and, if it is, whether the growth and replication rate can exceed the rate of processes causing loss of viability and death, resulting in net population increase or, at the minimum, a state of viable dormancy. The factors that lead to stable population establishment in new isolated environments have been considered for many decades and were famously synthesized in the work of MacArthur & Wilson [10] on island biogeography. The applicability of island biogeography and some of its ideas to the interplanetary (even interstellar) transfer of life has been considered before [11–13], but it has not been critically examined, and surprisingly, there has been no attempt to apply its principles to planetary protection. In this paper, we use island biogeography to reconsider the probabilistic approach to planetary protection, proposing establishment of a population as the primary criterion.

## 2. Theory

### 2.1. Island biogeography

MacArthur & Wilson’s [10] *The Theory of Island Biogeography* unites ideas in both biogeography and ecology to give quantitative estimations for inter-island population dynamics [10]. The credo of the theory is that the number of species on an island is determined by both the size of the island and its distance from the mainland or other islands. The balance between these factors provides a dynamic equilibrium of immigration I and extinction ϵ, such that one has a net population size with a given number of species S*,*


(2.1) $$\frac{\text{d}S}{dt}=I-E.$$

**Figure 1.**
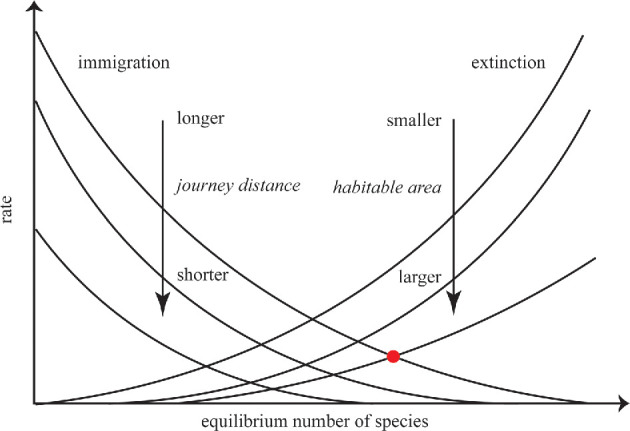
Equilibrium model for multiple planets separated by different journey times from a planet with a productive biosphere and different habitable area sizes. The intersection of the immigration and extinction curves gives the average number of species present on the colonized planet. The red point indicates the maximal species situation. This figure assumes that new species are not being created by speciation events and only by the balance of immigration and extinction. Adapted from [10].

The step of viewing planets as analogous to islands in terms of biogeography is a simple one; both planets and islands are physical locations with a relatively high degree of separation, and in both cases, there are phenomena that can carry small physical objects between them. As locations, they may both have habitable and uninhabitable environments for specific biota. Thus, on face value, equilibrium theory would be applicable to understanding interplanetary population dynamics. In table 1, we illustrate comparisons between the island biogeography theory and the interplanetary application, focusing on the physical analogies that control the rate of immigration of organisms. In figure 1, we revise the classic result from MacArthur & Wilson [10] for speciation in a multiplanet system.

**Table 1.** Planets as islands—a comparison in biogeography.

	physical systems		
biogeography mechanism	terrestrial islands	planetary islands	effect on population
dispersal mechanism	prevailing winds; ocean currents; self-propelled travel	gravitational potentials; gravitational perturbations; rock/dust fields; tidal evolution	increase/decrease immigration rate
dispersal filter	mountain ranges; island separation	interplanetary journey time (distance); atmospheric re-entry; shock pressure and temperature during ejection	reduces immigration rate
changing geophysical dynamics	plate tectonic shift; climatic changes	gravitational drift; plate tectonic shift; climatic changes	over long times can increase or decrease immigration rate

One aspect where island biogeography for planets breaks down is that, unlike small Earth islands, propagules arriving on a planet generally have a lower probability of landing near previous immigrants due to the vast planetary surface area, scaling with the square of the planetary radius. For most realistic scenarios, this would reduce the effective immigration rate to the point where it can be considered irrelevant in the equilibrium population. For Earth, assuming propagules randomly fall across the surface, the chance that any two land within 10 km is about $10^{-6}$.

**Figure 2.**
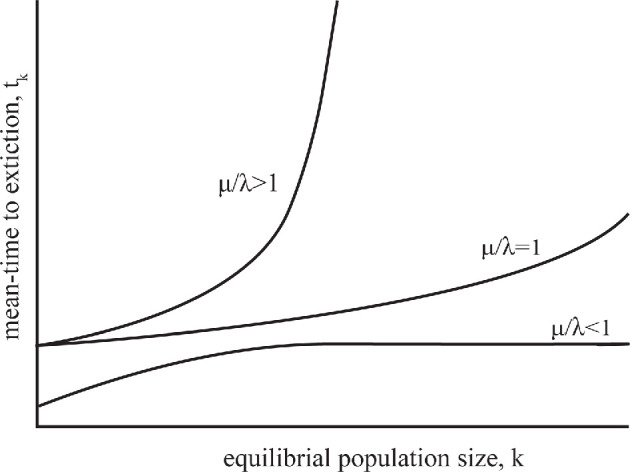
Mean-time to extinction curves for different birth–death rate ratios with population size. For μ/λ≥1, $t_{k}$ is unbounded. Note that to be consistent with the notation in microbiology with growth as μ, we denote death rates by λ, opposite to that of MacArthur and Wilson, where they use λ and μ as the rates of birth and death, respectively.

MacArthur and Wilson define successful colonization by a long mean-time to extinction for an immigrant population. This idea is the basis for our suggestions on planetary protection. Let μ and λ denote the birth and death rates per unit time of a single-species population, respectively. According to their model, the mean-time to extinction $t_{k}$ for an equilibrium population of size k is approximately μ/(μ−λ). This assumes that the population is free to grow until reaching some maximum size, determined by the environment through its resources, known as the environment carrying capacity, k. In figure 2, we illustrate from [10] how the birth–death rate ratio μ/λ can limit the mean-time to extinction. There are other models of population growth but that of MacArthur and Wilson comes from first principles such that these concepts are the general case in most models [14,15].

MacArthur and Wilson’s original theory was developed specifically for terrestrial island ecosystems. Consequently, we cannot directly apply their framework to planetary-scale environments without appropriate modifications. For example, although the same power-law relation for species with area, i.e. $S∼A^{constant}$, has been found for both microbes and mammals, a study found that microbial speciation was more strongly driven by environmental heterogeneity [16]. Our framework begins with the analogy between planets and islands, which we argue gives good grounds to expect the general trends of immigration and extinction rates with area and distance to remain. What we develop more specifically is the theory for the mean-time to extinction, as this can be applied to microbial species.

Also of importance is that we are considering the transfer of microorganisms through human agency, which is different from the natural processes originally considered by MacArthur and Wilson. The introduction of microorganisms to other planetary bodies, in the case of planetary protection concerns, is linked to spacecraft construction, deployment and exploration. On Earth, anthropologists and archaeologists have, for decades, examined analogous processes using island biogeography, assessing how human-mediated settlement impacts previously pristine ecosystems on Earth. These historical and archaeological studies document extensive ecological transformations, including numerous extinctions and substantial alterations to native species composition resulting from both intentional and accidental introductions of biota [17–22]. However, whether these same concerns, which have largely focused on macrofauna, apply to microbial transfer across interplanetary space is the question that must be addressed.

### 2.2. Microbial birth and death rates

**Figure 3.**
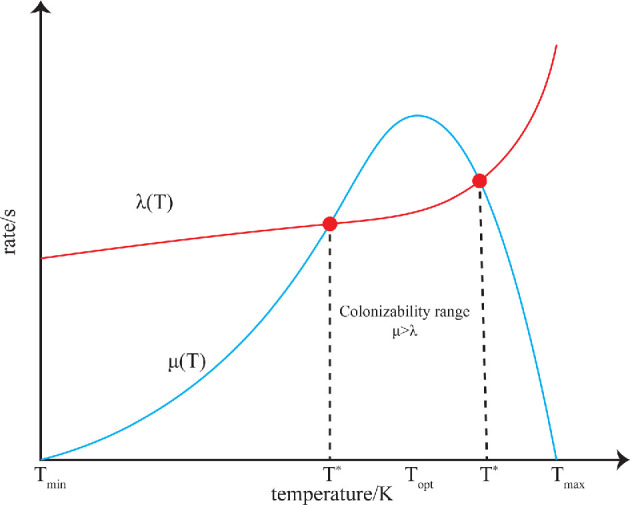
Birth (blue line) and death (red line) rates as a function of temperature. Red points mark the zero net population growth thresholds, where the death rate becomes higher than the birth rate, which results in eventual extinction.

To illustrate how environmental extremes influence the birth–death rate and thus colonization success, we can choose any parameter relevant to microbial growth. For example, consider temperature and how it may bound $\frac{\mu }{\lambda }>1$, necessary for an unbounded mean-time to extinction. The general functional form for the thermal growth (birth) rate has been well studied (see [23] for a review). We consider a generalized form describing the effect of temperature on microbial growth over the entire range:


(2.2) $$\mu (T)=ATe^{-\frac{a}{T}}\left(1-e^{b\left(T-T_{\max}\right)}\right),$$

where T is temperature, {A,a,b} are a set of real coefficients and $T_{\max}$ is the first highest temperature at which the birth rate is zero [24]. The death rate with temperature has not been as well studied, and there are no theoretical models available for the complete temperature range. Here, we use an approximation for the sake of demonstrating the conceptual idea (figure 3), but we can physically motivate some possible functional forms.

Although vitrification may improve survival at low temperatures, organisms still lose viability [25], so there is always background death at any temperature. Below the minimum temperature for birth rate, $T_{\min}$, extinction is inevitable. Therefore, death rates might be approximately constant or increase monotonically as temperature decreases. At high temperatures, thermal inactivation kinetics and Arrhenius-type reactions dominate, leading to deaths; thus, an exponential dependence is expected. In the mid-range, growth processes dominate, making the death rate comparatively negligible and its variation unclear. Various functional forms satisfy these constraints, but generally one would expect

(2.3) $$\lambda (T)∼f(T)\cdot g(T,T_{opt},T_{\min})+{\text{e}}^{\beta T},$$

where f,g are functions that take care of low temperature and intermediate regions, respectively.

Planetary protection concerns the points where $\mu (T^{*})=\lambda (T^{*})$, with $T^{*}$ being the equilibrium temperature above or below which the population eventually becomes extinct (figure 3). These transition regions are not commonly investigated since disentangling microbial death and birth is experimentally challenging [26]. Generally, microbiologists measure the lag phase and doubling time when quantifying microbial populations. However, these measurements, although sometimes simple to make, are a combination of the underlying metabolic and physiological factors that lead to microbial replication and the factors that are leading to loss of microbial viability and death, whereby a net population increase observed in an experiment occurs when the former dominates over the latter. As we discuss here, since the functions that describe the factors that lead to replication and those that lead to loss of viability and death are not necessarily simple or correlated to one another, ideally one would disentangle them in order to be able to both understand the biochemical basis of the net population growth and model it for predictive purposes.

Cells can also enter a dormant state, remaining viable but ceasing replication. If relocated or conditions change, they may replicate again, posing concerns for planetary protection. There is no universal set of physiological requirements that define dormancy [27], but if considered as a balance between maintenance processes and loss of viability, dormancy corresponds to cells near the intersection of birth and death rates, especially at low temperatures (see figure 3) and/or limitations of energy or nutrients. Again, a grasp of the mathematical functions of the birth and death curves could allow us to predict the intersections of those curves and thus where these borderline dormancy states may lie.

### 2.3. Rethinking successful colonization

Defining *success* for a planetary protection strategy depends on whether one is concerned with the contamination of scientific results or of other planets’ environments, or both. For the former, a probabilistic framework is appropriate as only the transfer process and the chemical integrity of the contaminant matter are appropriate [5]. Since we can never be certain what microbes may be on a spacecraft, a key initial inquiry should be: Can any known microbe achieve prolonged dormancy or net population growth in the destination conditions? If yes, one would make a probabilistic assessment of its transfer likelihood; otherwise, such assessment is not warranted. Thus, probability is relevant until the point of arrival on the extraterrestrial body. Methods to calculate probabilities, such as stochastic binomial models, were originally designed for evaluating spacecraft sterilization procedures [28], and not for predicting successful colonization or population establishment in new environments. We illustrate the difference by considering the phase space of the mean-time to extinction function.

**Figure 4.**
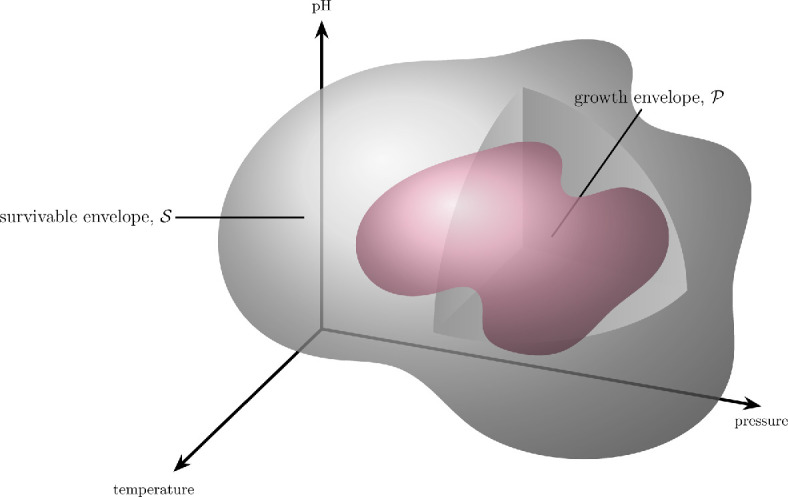
Three-dimensional example phase space for the mean-time to extinction growth threshold. Purple sub-space represents the parameter space beyond which the mean-time to extinction is too short for colonization. Grey sub-subspace is the habitable zone, beyond which the organism will die.

We define successful colonisation as


(2.4) $$t_{k}\geq t_{c},$$

where $t_{c}$ is a critical time beyond which the species is considered established—this could be the planet’s habitable lifetime $t_{hab}$ or a mission timeframe. The mean-time to extinction is a multivariate function


(2.5) $$t_{k}=t_{k}(T,p,\text{pH},k,\lambda ,\mu ,M,x),$$

where T,p,pH are the temperature, pressure, and acidity of the environment with carrying capacity k, M is the mutation rate and x is the initial population size. Determining $t_{k}$ is a highly multidimensional, nonlinear problem since microbes are capable of rapid reproduction, short-term physiological adaptation, and beneficial genetic alterations (e.g. horizontal gene transfer). Consider a d-dimensional phase space where each dimension corresponds to a parameter in $t_{k}$. Restricting $t_{k}$ to three dimensions for simplicity, the phase space is


(2.6) $$\begin{array}{lcr} & P=\left\{(T,p,\mathrm{pH})\in {\mathbb{R}}^{3}|t_{k}(T,p,\mathrm{pH})\geq t_{c}\right\}, & \end{array}$$

which defines a geometric surface beyond which colonization is not possible (figure 4). Within the survivability envelope lies the space for net population growth. A microbe might initially *survive* but not *replicate*. The idea to tackle these issues using concepts from dynamical systems theory has, as far as we are aware, not before been considered. In the next section, we elucidate further how these spaces help us see colonizability as binary.

Microbiological life can establish populations without going extinct in extreme conditions far outside those *optimal* for growth. For example, cyanobacteria in extreme cold environments like the Arctic and Antarctic have a higher optimal growth temperature than their habitat [29]. Despite slow growth, their mean-time to extinction is large, so the population is well-established. Thus, although growth is small, μ/λ>1 remains. A small body of literature seeks to define these phase spaces for microbiology, but data are limited [30–32].

Various non-environmental and non-species-specific mathematical models provide a mean-time to extinction function [10,33–36]. These could be used to construct parametrized models from experimental data on how environmental parameters influence growth and death. This task is complex as models have varied functional forms, e.g. exponential versus power-law relationships for environmental versus demographic stochasticity [35]. These functions depend on carrying capacity, which fluctuates and is driven by complicated processes (e.g. [31]). All these features vary between planetary environments.

## 3. Planetary protection: a new approach

Currently, planetary protection risk is assessed through an estimated ‘probability for growth’, $P_{g}$. However, successful growth of an organism is not probabilistic; either the birth rate exceeds the death rate, leading to a net population increase, or it does not. Similarly, at the intersection of these rates, conditions either allow for prolonged dormancy over some defined time period or they do not. Thus, a simpler approach is to consider whether the environment lies outside the colonizability domain (short mean-time to extinction) for the microorganism’s phase space. Each species has a definitive survivable envelope outside of which $P_{g}=0$ as the organism is dead, but within which there is a zone defined by parameters sufficient for dormancy or replication to maintain or establish a population (long mean-time to extinction), where $P_{g}=1$.

These spaces define a binary option: either the planetary conditions permit colonization or they do not. At extremes, we already consider the survival of an organism to be binary; beyond certain temperature thresholds on spacecraft surfaces, we conclude no life can survive. Conversely, in locations with liquid water, $P_{g}$ is typically set to unity [8], as the presence of liquid water is sometimes assumed (erroneously) to imply habitable conditions. Many other variables, such as salinity and pressure, define the edges of survival and microbial growth/reproduction. A probabilistic argument is misleading, as it implies that with a sufficiently large $n_{0}$, if $P_{g}>0$ (which presently seems arbitrarily assumed [8]), some proportion of the contaminant organisms will survive *and* establish a population. For microbes outside their optimal growth conditions, the probabilistic approach may be misleading since even outside the optimum, $P_{g}$ is simply 1 no matter how slow growth is.

The generally non-probabilistic growth of microorganisms is evident in the science of microbiology. If growth under specific conditions were probabilistic, then since the emergence of microbiology in the seventeenth century, microbiologists would have empirically created tables of growth probabilities in given medium formulations. Every time they wished to grow an organism, they would consult these tables and prepare a given number of flasks to ensure that one or two of them had successful growth. However, this is not how the science operates. Instead, they use a single medium; if growth fails, they assume conditions are unsuitable and try different media. This reflects a largely binary problem.

This is because reproduction cannot partially occur; a cell either divides or it does not. Division depends on whether nutrients, energy, physico-chemical conditions, inter alia, are sufficient for replication. Net population growth depends on the replication rate exceeding the death rate. Stochastic events (e.g. local variations in conditions or the metabolic state of a cell operating at its thermodynamic limits) may introduce some probability into successful growth, especially with small initial populations. Probabilistic models can also describe exit from dormant states like bacterial spores [37], predicting the number of spores that germinate. While we acknowledge probabilistic models, we suggest that once an actively metabolizing cell is in an environment, a single probability term for successful colonization is inadequate. It seems parsimonious to assume that if conditions support growth, stochastic events will not prevent it, allowing us to consider the mean-time to extinction based on birth and death rates to assess colonization. Our approach, in *assuming* that microbial life will reach extraterrestrial bodies, is arguably also a more cautious one. These arguments apply to assessing whether environmental and energetic conditions allow for the persistence of dormant cellular states over some defined time period.

For planetary protection, we propose an alternative procedure aiming to determine whether the mean-time to extinction is shorter than a reasonable timescale (e.g. mission or exploration lifetime); if so, no planetary protection risk is concluded. Detailed environmental studies must evaluate parameter ranges for key microbiological variables (e.g. temperature, pressure, acidity, medium content), indicating which microorganisms find the environment habitable. We then determine whether μ≥λ for given planetary parameters, in which case colonization is inevitable. If μ<λ, we need to estimate experimentally the mean-time to extinction to see if it is long enough to be considered colonization, i.e. similar to geophysical timescales or longer than mission windows.

For many microbes, birth and death rate data are unknown, even for single stressors like temperature. On planets like Mars, numerous stressors—salinity, pressure, radiation exposure, perchlorate toxicity [38,39]—create different genetic pressures on microorganisms. Therefore, we need a theoretical method, constrained by experiments, to estimate the mean-time to extinction for populations on other celestial bodies. Planetary protection research cites many knowledge gaps, but this issue has not been previously identified.

### 3.1. Problem of measuring microbial death

Death in microbes, particularly at low temperatures, can be challenging to define. In the absence of replication, dormant states may still allow sufficient metabolic activity to overcome inescapable background rates of detrimental processes such as racemization of amino acids, depurination of DNA and thermal degradation over time [40]. Yet, there is always a background level of population decline, albeit potentially very slow. This is consistent with the MacArthur and Wilson model: for μ/λ<1*,* the mean-time to extinction does not scale indefinitely with population size, though it may still be long, set by the doubling time, yet death remains present.

While birth and death rates both depend on cellular metabolism, they must be isolated to model them effectively and determine which processes, metabolic, environmental or otherwise, define the birth curve (i.e. processes that maintain a cell in a viable state and potentially allow for replication) or lead to death (i.e. processes that cause loss of viability) [41]. More in-depth studies of metabolic and bioenergetic pathways, molecular biology and the interactions of cells with their environments could allow for better quantification of these curves and thus lead to better predictive models under stated environmental conditions. Most experiments are conducted under fixed laboratory conditions, but environments like Mars present diurnal temperature fluctuations [42] that introduce stochasticity into population dynamics. These temporal and environmental changes must be considered when assessing microbial population dynamics [43]. Stochasticity in genome-expressed pathways makes predictions extremely challenging. However, with sufficient computing resources and more metabolic data, it should be possible to combinatorially check gene combinations to assess optimal situations, similar to [44].

### 3.2. Colonizability: an example

**Figure 5.**
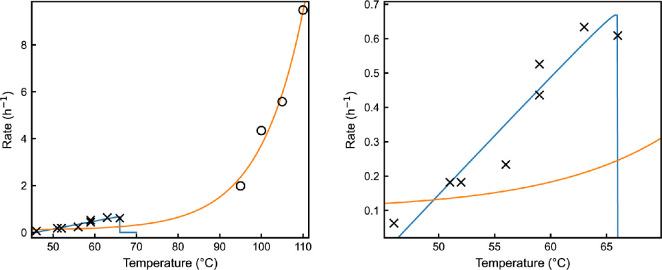
Left: growth rate of *Geobacillus stearothermophilus* as a function of temperature from [45] (black crosses), fitted with the Cardinal model (blue line). Thermal death rate (open black circles) from [46] fitted with an Arrhenius-type model (orange line). Right: same plot as on the left, enlarged to show intersections of the death and growth rate curves. Either side of the intersections, the mean-time to extinction would be finite and will not positively co-vary with the population’s growth rate.

Currently, we lack separate replication and death rate curves across full temperature ranges. For example, figure 5 presents the growth rate of the thermophile *Geobacillus stearothermophilus* as a function of temperature from [45]. While growth rates are measured during the exponential phase—where death rates are usually considered negligible—this assumption breaks down near critical temperatures ($\mu (T^{*})=\lambda (T^{*})$). In these regions, growth rate measurements may not accurately reflect replication rates due to significant death rates. By including a crude death rate curve following MacArthur and Wilson’s approach, we highlight the uncertainties in extrapolating to low temperatures with limited data. This illustrates how replication and death processes are distinct and can lead to different mean times to extinction near critical temperatures, even when resources are sufficient. Clearly, without separate models for replication and death processes, our ability to make accurate predictions is limited, especially at the intersections of these curves, near which cells may experience dormant states.

**Figure 6.**
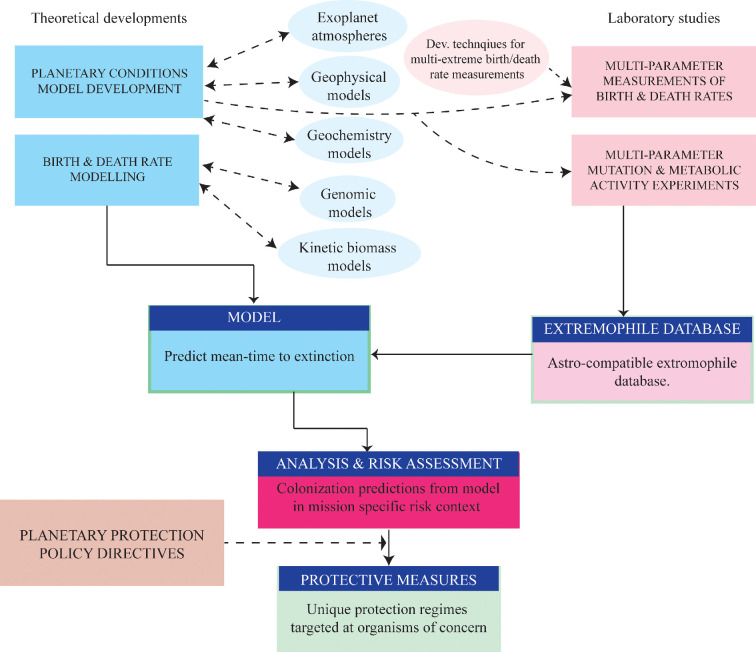
A roadmap to a new planetary protection framework.

Theoretical and laboratory experiments should eventually enable us to predict whether a microbe with specific characteristics would exceed a certain mean-time to extinction under specific planetary conditions. Alternatively, since we never know the full inventory of organisms on a spacecraft, one could collect physical and chemical data from an extraterrestrial environment and develop a database of known organisms capable of achieving net population growth or dormancy under those conditions. This *extremophile database* would be useful for two reasons. First, it defines organisms of concern—if they are mission contaminants, they could colonize or persist in the destination environment. Second, these organisms could be used for deliberate introduction to a planetary environment one aims to inoculate or terraform. It may also be possible to use theoretical considerations, such as environmental energy availability, to predict which organisms might achieve net population growth [47,48]. Figure 6 illustrates a roadmap for developing such a framework with practical aims for the planetary protection community. Such predictive capabilities, linked to policy, already exist in the food safety community, where laboratory experiments and theoretical models predict microbial contamination [49,50]. Our suggestions are summarized in table 2, including specific techniques.

**Table 2.** Planetary protection programme of microbial research, including techniques.

	objective	techniques	references
modelling development	microbial colonization models based on mean-time to extinction, incorporating environmental stressors	Arrhenius-based models for microbial growth and death rates under extreme conditions, phase-space diagrams to visualize colonization limits	[23,49]
	death rate models for microbial populations under multiple stressors	kinetic inactivation models	[46,51]
	create extremophile database cataloguing survival profiles across diverse planetary environments	multivariate extinction models, parameterize theoretical models	[51,52]
	dynamic model development	stochastic factors such as diurnal temperature fluctuations	[42,52]
laboratory studies	measurement of microbial birth and death rates across stressors range (e.g. temperature, pressure, acidity)	continuous culture techniques, fluorescence microscopy	[23,25]
	measurement of microbial death processes (e.g. apoptosis) in extreme planetary environments using simulations of e.g. Mars-like conditions	environmental chambers, real-time high-content live-cell imaging	[26,31,38,53]
	bioenergetics studies of microbial replication and death under stress to distinguish between survival and reproductive processes	metabolomics, mass spectrometry, respirometry, time-lapse microscopy	[41]
	validation of colonization thresholds for various microbes using extreme environment simulations to estimate the mean-time to extinction	Mars simulation chambers, environmental chambers, phase-space analysis	[26,30,39]

## Data Availability

The growth rate data presented in figure 5 were provided to us on request by the author of the original study [45]. The death rate data in figure 5 were taken directly from table 2 in the paper [46].

## References

[1] Clark BC. 2001 Planetary interchange of bioactive material: probability factors and implications. Orig. Life Evol. Biosphere **31**, 185–197. (10.1023/A:1006757011007) 11296521

[2] Tepfer D. 2008 The origin of life, panspermia and a proposal to seed the universe. Plant Sci. **175**, 756–760. (10.1016/j.plantsci.2008.08.007)

[3] DeVincenzi D, Stabekis P, Barengoltz J. 1983 A proposed new policy for planetary protection. Adv. Space Res. **3**, 13–21. (10.1016/0273-1177(83)90167-9) 11538978

[4] Coustenis A *et al*. 2023 Planetary protection: an international concern and responsibility. Front. Astron. Space Sci. **10**, 1172546. (10.3389/fspas.2023.1172546)

[5] McCoy KJ, DiNicola M, Everline C, Burgoyne H, Reinholtz K, Clement B. 2021 Europa clipper planetary protection probabilistic risk assessment summary. Planet. Space Sci. **196**, 105139. (10.1016/j.pss.2020.105139)

[6] NASA. 2022 NASA Technical Standard: implementing planetary protection requirements for space flight. Baseline version. NASA.

[7] COSPAR Policy on Planetary Protection. 2023 COSPAR Panel on Planetary Protection.

[8] Kazarians GA *et al*. 2017 The evolution of planetary protection implementation on Mars landed missions. In 2017 IEEE Aerospace Conf, *Big Sky, MT, USA*. (10.1109/AERO.2017.7943576)

[9] Council NR. 2012 Assessment of planetary protection requirements for spacecraft missions to icy solar system bodies. Washington, DC: The National Academies Press.

[10] MacArthur RH, Wilson EO. 1967 The theory of island biogeography. Princeton, NJ: Princeton University Press.

[11] Lingam M, Loeb A. 2017 Enhanced interplanetary panspermia in the TRAPPIST-1 system. Proc. Natl Acad. Sci. USA **114**, 6689–6693. (10.1073/pnas.1703517114) 28611223 PMC5495259

[12] Cockell CS *et al*. 2007 Interplanetary transfer of photosynthesis: an experimental demonstration of a selective dispersal filter in planetary island biogeography. Astrobiology **7**, 1–9. (10.1089/ast.2006.0038) 17407400

[13] Cockell CS. 2008 The interplanetary exchange of photosynthesis. Orig. Life Evol. Biospheres **38**, 87–104. (10.1007/s11084-007-9112-3) 17906941

[14] Soulé ME (ed). 1987 Viable populations for conservation, pp. 1–10. Cambridge, UK: Cambridge University Press.

[15] Mangel M, Tier C. 1993 A simple direct method for finding persistence times of populations and application to conservation problems. Proc. Natl Acad. Sci. USA **90**, 1083–1086. (10.1073/pnas.90.3.1083) 11607362 PMC45815

[16] Horner-Devine MC, Lage M, Hughes JB, Bohannan BJM. 2004 A taxa–area relationship for bacteria. Nature **432**, 750–753. (10.1038/nature03073) 15592412

[17] Finney BR. 1988 Solar system colonization and interstellar migration. Acta Astronaut. **18**, 225–230. (10.1016/0094-5765(88)90103-8)

[18] Finney BR, Jones EM. 1985 Voyagers into ocean space. In Interstellar migration and the human experience (eds RF Ben, MJ Eric), pp. 164–179. Berkeley, CA: University of California Press.

[19] Keegan WF, Diamond JM. 1987 Colonization of islands by humans: a biogeographical perspective. In Advances in archaeological method and theory (ed. MB Schiffer), pp. 49–92. San Diego, CA: Academic Press. (10.1016/B978-0-12-003110-8.50005-0)

[20] Fitzpatrick SM. 2022 'Detritus of a coming world’: the colonization of islands as microcosms for human impacts on an interplanetary scale. In Speciesism in biology and culture: how human exceptionalism is pushing planetary boundaries (eds B Swartz, BD Mishler), pp. 65–93. Cham, Switzerland: Springer. (10.1007/978-3-030-99031-2_4)

[21] Leppard TP, Fitzpatrick SM. 2025 Island archaeology provides ecological and behavioral analogs for off-planet exploration and colonization. Futures **166**, 103544. (10.1016/j.futures.2025.103544)

[22] Holcomb JA, O’Leary BL, Fairén AG, Mandel RD, Wegmann KW. 2024 The emerging archaeological record of Mars. Nat. Astron. **8**, 1490–1492. (10.1038/s41550-024-02439-w)

[23] Noll P, Lilge L, Hausmann R, Henkel M. 2020 Modeling and exploiting microbial temperature response. Processes **8**, 121. (10.3390/pr8010121)

[24] Ratkowsky DA, Lowry RK, McMeekin TA, Stokes AN, Chandler RE. 1983 Model for bacterial culture growth rate throughout the entire biokinetic temperature range. J. Bacteriol. **154**, 1222–1226. (10.1128/jb.154.3.1222-1226.1983) 6853443 PMC217594

[25] Clarke A, Morris GJ, Fonseca F, Murray BJ, Acton E, Price HC. 2013 A low temperature limit for life on earth. PLoS One **8**, e66207. (10.1371/journal.pone.0066207) 23840425 PMC3686811

[26] Wu R *et al*. 2024 Bacterial killing and the dimensions of bacterial death. NPJ Biofilms Microbiomes **10**, 87. (10.1038/s41522-024-00559-9) 39289404 PMC11408613

[27] McDonald MD *et al*. 2024 What is microbial dormancy? Trends Microbiol. **32**, 142–150. (10.1016/j.tim.2023.08.006) 37689487

[28] Fredrickson AG. 1966 Stochastic models for sterilization. Biotechnol. Bioeng. **8**, 167–182. (10.1002/bit.260080114)

[29] Tang EPY, Tremblay R, Vincent WF. 1997 Cyanobacterial dominance of polar freshwater ecosystems: are high‐latitude mat‐formers adapted to low temperature? J. Phycol. **33**, 171–181. (10.1111/j.0022-3646.1997.00171.x)

[30] Harrison JP, Gheeraert N, Tsigelnitskiy D, Cockell CS. 2013 The limits for life under multiple extremes. Trends Microbiol. **21**, 204–212. (10.1016/j.tim.2013.01.006) 23453124

[31] Qiu Y, Zhou Y, Chang Y, Liang X, Zhang H, Lin X, Qing K, Zhou X, Luo Z. 2022 The effects of ventilation, humidity, and temperature on bacterial growth and bacterial genera distribution. Int. J. Environ. Res. Public Health **19**, 15345. (10.3390/ijerph192215345) 36430064 PMC9691097

[32] Harrison JP, Dobinson L, Freeman K, McKenzie R, Wyllie D, Nixon SL, Cockell CS. 2015 Aerobically respiring prokaryotic strains exhibit a broader temperature–pH–salinity space for cell division than anaerobically respiring and fermentative strains. J. R. Soc. Interface **12**, 20150658. (10.1098/rsif.2015.0658) 26354829 PMC4614477

[33] Thakur B, Meyer-Ortmanns H. 2023 Controlling the mean time to extinction in populations of bacteria. Entropy **25**, 755. (10.3390/e25050755) 37238510 PMC10217645

[34] Wylie CS, Shakhnovich EI. 2012 Mutation induced extinction in finite populations: lethal mutagenesis and lethal isolation. PLoS Comput. Biol **8**, e1002609. (10.1371/journal.pcbi.1002609) 22876168 PMC3410861

[35] Lande R. 1993 Risks of population extinction from demographic and environmental stochasticity and random catastrophes. Am. Nat. **142**, 911–927. (10.1086/285580) 29519140

[36] Ovaskainen O, Meerson B. 2010 Stochastic models of population extinction. Trends Ecol. Evol. **25**, 643–652. (10.1016/j.tree.2010.07.009) 20810188

[37] Barker G, Malakar P, Peck M. 2005 Germination and growth from spores: variability and uncertainty in the assessment of food borne hazards. Int. J. Food Microbiol. **100**, 67–76. (10.1016/j.ijfoodmicro.2004.10.020) 15854693

[38] Matarredona L, Camacho M, Zafrilla B, Bonete MJ, Esclapez J. 2020 The role of stress proteins in haloarchaea and their adaptive response to environmental shifts. Biomolecules **10**, 1390. (10.3390/biom10101390) 33003558 PMC7601130

[39] Archer PD. 2019 Perchlorate on Mars — overview and implications. In 9th Int. Conf. on Mars, 22–25 July 2019, Pasadena, CA, USA.

[40] Price PB, Sowers T. 2004 Temperature dependence of metabolic rates for microbial growth, maintenance, and survival. Proc. Natl Acad. Sci. USA **101**, 4631–4636. (10.1073/pnas.0400522101) 15070769 PMC384798

[41] Bae SY, Guan N, Yan R, Warner K, Taylor SD, Meyer AS. 2020 Measurement and models accounting for cell death capture hidden variation in compound response. Cell Death Dis. **11**, 255. (10.1038/s41419-020-2462-8) 32312951 PMC7171175

[42] Atri D, Abdelmoneim N, Dhuri DB, Simoni M. 2022 Diurnal variation of the surface temperature of Mars with the Emirates Mars mission: a comparison with curiosity and perseverance rover measurements. Mon. Not. R. Astron. Soc. **518**, L1–L6. (10.1093/mnrasl/slac094)

[43] Huete-Stauffer TM, Arandia-Gorostidi N, Díaz-Pérez L, Morán XAG. 2015 Temperature dependences of growth rates and carrying capacities of marine bacteria depart from metabolic theoretical predictions. FEMS Microbiol. Ecol. **91**, fiv111. (10.1093/femsec/fiv111) 26362925

[44] Sun D, Liu Y, Zhang XS, Wu LY. 2018 CEA: combination-based gene set functional enrichment analysis. Sci. Rep. **8**, 13085. (10.1038/s41598-018-31396-4) 30166636 PMC6117355

[45] Molinaro C, Bénéfice M, Gorlas A, Da Cunha V, Robert HML, Catchpole R, Gallais L, Forterre P, Baffou G. 2022 Life at high temperature observed in vitro upon laser heating of gold nanoparticles. Nat. Commun. **13**, 5342. (10.1038/s41467-022-33074-6) 36097020 PMC9468142

[46] Ayeni AO, Samuel IT, Adekeye BT, Agboola O, Nwinyi OC, Oladokun O, Ayoola AA, Elehinafe FB. 2022 Inactivation kinetics and thermodynamics assessments of Geobacillus stearothermophilus during thermal sterilization for products safety. South Afr. J. Chem. Eng. **42**, 223–228. (10.1016/j.sajce.2022.09.003)

[47] Rogers KL, Amend JP, Gurrieri S. 2007 Temporal changes in fluid chemistry and energy profiles in the Vulcano Island hydrothermal system. Astrobiology **7**, 905–932. (10.1089/ast.2007.0128) 18163870

[48] Hoehler TM. 2007 An energy balance concept for habitability. Astrobiology **7**, 824–838. (10.1089/ast.2006.0095) 18163865

[49] Leporq B, Membré JM, Dervin C, Buche P, Guyonnet JP. 2005 The ‘Sym’Previus’ software, a tool to support decisions to the foodstuff safety. Int. J. Food Microbiol. **100**, 231–237. (10.1016/j.ijfoodmicro.2004.10.006) 15854708

[50] Membre J, Leporq B, Vialette M, Mettler E, Perrier L, Thuault D, Zwietering M. 2005 Temperature effect on bacterial growth rate: quantitative microbiology approach including cardinal values and variability estimates to perform growth simulations on/in food. Int. J. Food Microbiol. **100**, 179–186. (10.1016/j.ijfoodmicro.2004.10.015) 15854703

[51] Hanson CA, Fuhrman JA, Horner-Devine MC, Martiny JBH. 2012 Beyond biogeographic patterns: processes shaping the microbial landscape. Nat. Rev. Microbiol. **10**, 497–506. (10.1038/nrmicro2795) 22580365

[52] Martiny JBH *et al*. 2006 Microbial biogeography: putting microorganisms on the map. Nat. Rev. Microbiol. **4**, 102–112. (10.1038/nrmicro1341) 16415926

[53] Gelles JD, Chipuk JE. 2016 Robust high-throughput kinetic analysis of apoptosis with real-time high-content live-cell imaging. Cell Death Dis. **7**, e2493. (10.1038/cddis.2016.332) 27906190 PMC5261025

